# Decoding the role of chromatin architecture in development: coming closer to the end of the tunnel

**DOI:** 10.3389/fpls.2014.00374

**Published:** 2014-08-21

**Authors:** Chongyuan Luo, Juan Dong, Yi Zhang, Eric Lam

**Affiliations:** ^1^Department of Plant Biology and Pathology, Rutgers the State University of New JerseyNew Brunswick, NJ, USA; ^2^The Waksman Institute of Microbiology, Rutgers the State University of New JerseyPiscataway, NJ, USA

**Keywords:** chromatin, chromatin beacons, chromatin conformation capture, INTACT method, cell fate specification

## Abstract

Form and function in biology are intimately related aspects that are often difficult to untangle. While the structural aspects of chromatin organization were apparent from early cytological observations long before the molecular details of chromatin functions were deciphered, the extent to which genome architecture may impact its output remains unclear. A major roadblock to resolve this issue is the divergent scales, both temporal and spatial, of the experimental approaches for examining these facets of chromatin biology. Recent advances in high-throughput sequencing and informatics to model and monitor genome-wide chromatin contact sites provide the much-needed platform to close this gap. This mini-review will focus on discussing recent efforts applying new technologies to elucidate the roles of genome architecture in coordinating global gene expression output. Our discussion will emphasize the potential roles of differential genome 3-D structure as a driver for cell fate specification of multicellular organisms. An integrated approach that combines multiple new methodologies may finally have the necessary temporal and spatial resolution to provide clarity on the roles of chromatin architecture during development.

## INTRODUCTION

Chromatin is the *de facto* genetic material of eukaryotic cells, which is composed of an extraordinarily complex array of nucleoprotein components. Numerous studies in the past decades have suggested that the transcription activity across the genome can be correlated with a number of microscopic features, such as chromatin compactness (Beisel and Paro, 2011), proximity of the locus to the nuclear laminar, nuclear pore or nucleolus (Misteli, 2007; Berman et al., 2011), and the mobility of chromatin (Chuang et al., 2006; Rosin et al., 2008). However, it has been difficult to unambiguously integrate microscopic data of chromatin behavior with the mechanisms of transcription regulation and epigenetic control. The recent advent of sequencing-based genome-wide chromosome conformation capture techniques (such as 3C or Hi-C) provided the much-needed tools to relate long-range chromatin interactions with other genomic and epigenomic features (Dekker et al., 2002; Lieberman-Aiden et al., 2009). However, the structural basis of 3C and Hi-C is not completely understood. In particular, it remains unresolved whether the chromatin interactions detected by 3C or Hi-C reflected molecular (10–100 nm) or cytological (100–1000 nm) proximity (Belmont, 2014).

Complement to the ligation-based proximity assays, tagging of genomic loci with chromatin beacons provides the means of measuring chromatin dynamics and spatial relevance to ultra-structures (Heun et al., 2001; Kato and Lam, 2001; Lam et al., 2004; Rosin et al., 2008). For studying chromatin conformation in polyploid plant species or endoreduplicated plant cells, this approach can enable the tracking of highly homologous alleles as shown in studies using mapped transgenic *Arabidopsis thaliana* plants (Rosin et al., 2008; Rosa et al., 2013). However, the introduction of chromatin beacons may associate with potential caveats, such as the large transgene construct can be targeted by gene silencing machinery in some cases and therefore alter the local chromatin context (Watanabe et al., 2005; Jovtchev et al., 2008, 2011).

In this mini-review, we will first summarize the global features revealed by Hi-C assays, followed by describing possible biological roles of chromatin architecture in cellular differentiation and during development. We will focus on a number of genetic studies involving genes encoding chromatin modifiers and conclude with perspectives of deploying cell-type-specific techniques to better resolve the functional consequence of the chromatin interactome.

## GLOBAL PATTERNS OF CHROMATIN ORGANIZATION

**FIGURE 1:**
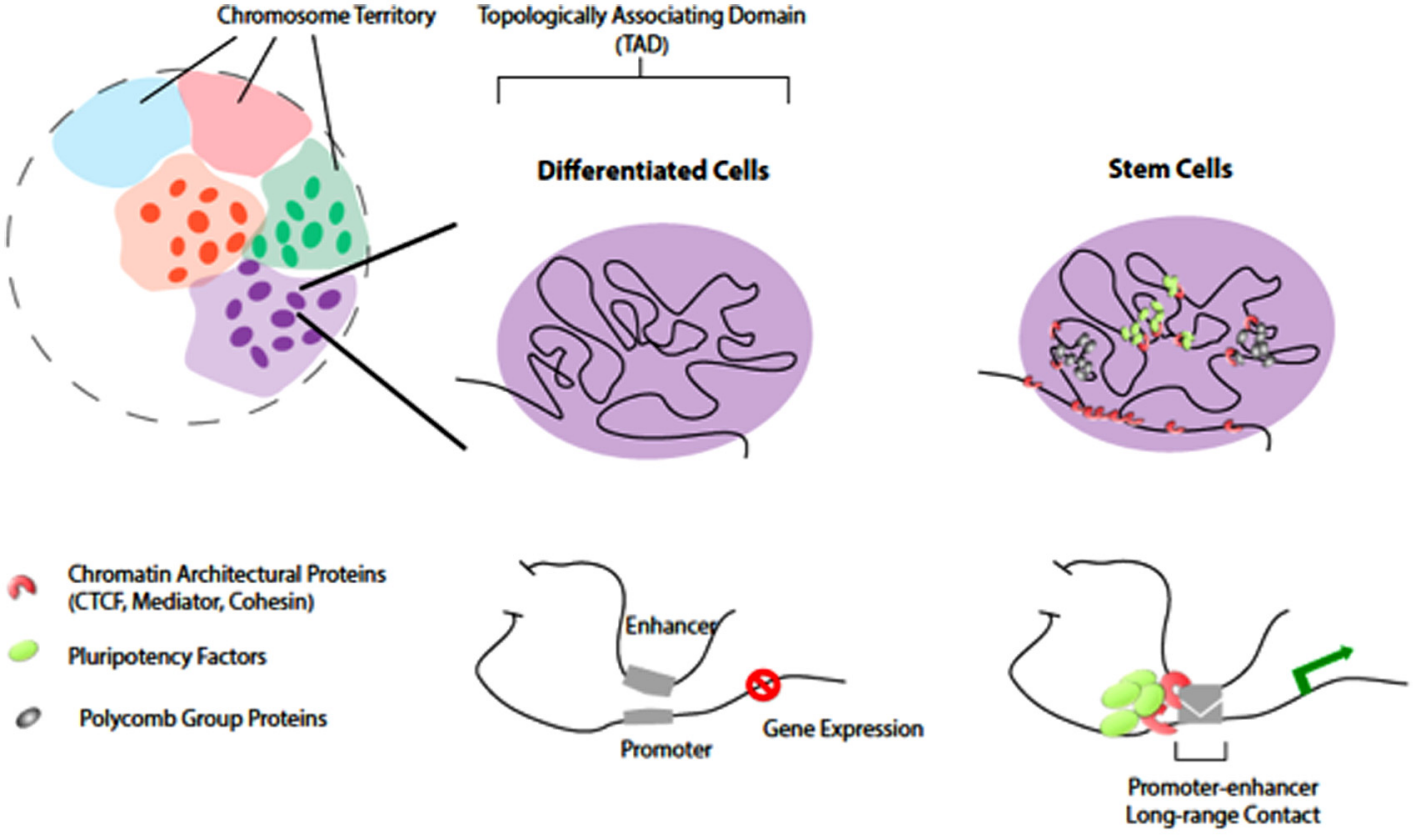
**Chromatin organization and topological domains.** Chromosomes occupy distinct territories in the nucleus (shaded with different colors). Long-range chromatin looping, at the sub-megabase level, partitions the chromosomal region into Topologically Associating Domains (TADs; individuals illustrated on the rights. The TADs remain largely unchanged in differentiated cells and stem cells. However, at a finer levH pluripotency factors and chromatin architectural proteins organize higher-order chromatin connectivity during reprogramming. Pluripotency factors co-localize and occupy distinct spatial regions from PcG proteins in stem cells. Such chromatin reorganization induced by pluripotency factors is important for cell-specific gene expression.

Recent Hi-C experiments performed with animal cells (Lieberman-Aiden et al., 2009; Dixon et al., 2012; Sexton et al., 2012) have confirmed the existence of chromosome territories that were previously described cytologically (**Figure 1**). The probabilities of intra-chromosomal interaction, even for loci that are 200 Mb apart, are much greater than that of inter-chromosomal interactions. This characteristic of Hi-C data has been utilized to assist genome-wide haplotype reconstruction and genome assembly scaffolding (Burton et al., 2013; Kaplan and Dekker, 2013; Selvaraj et al., 2013). In both insects and mammals that have been studied, the vast majority of the genome is organized into large topological domains with median size of ∼100 kb for *Drosophila* or ∼1 Mb for human. Considering the 20× difference between the *Drosophila* and human genome sizes, the scale of topological domains are largely comparable (Dixon et al., 2012; Sexton et al., 2012). Several types of genome compartmentalization mechanisms may be involved to create and maintain topological domains. For example, the binding of CTCF (CCCTC-binding factor, insulator binding protein) and a repressive histone mark H3K9me3 were found enriched at the boundary of topological domains (Dixon et al., 2012). Large scale genome features such as lamina-associated domains and late-replicating-chromatin were also found to partially overlap with topological domain boundaries, suggesting that nuclear ultra-structure and cell cycle control may contribute to the formation of such domains.

A striking finding from studies with both *Drosophila* and mammals was that topological domains appear to be stable across cell types and were also conserved between mouse and human (Dixon et al., 2012; Sexton et al., 2012). This finding suggests that the mechanism maintaining genome topological domains is likely ancient in the animal kingdom. With greater sequencing depth (150× coverage of the haploid genome) and improved data processing algorithms, chromatin interactions could be determined with a resolution of 5–10 kb for the human genome (Jin et al., 2013). Unlike the stable long-range topological domains, the observed short-range interactions are highly cell-type specific, presumably reflecting specific regulatory activities such as enhancer–promoter interactions. An intriguing finding was that perturbation of the cellular state with a signaling molecule did not significantly alter genomic patterns of short-range looping (Jin et al., 2013), which may indicate that enhancer–promoter interaction is integral to programming cellular identity and may be primed before the signaling event to facilitate timely cellular response.

In plants, global chromatin organization of *Arabidopsis thaliana* has been studied with 4C method using 13 viewpoints covering each chromosome and context (Grob et al., 2013). With this 4C dataset, the work showed that intra-arm interactions are 10× more abundant than inter-arm interactions, which are also about 10× more frequent than inter-chromosomal interactions. Consistent with the findings in human, inter-arm and inter-chromosomal interactions are more likely to occur between regions that are distant to the centromere, which are also gene rich and actively expressed. However, one exception to this trend is that strong interactions were observed between the heterochromatin knob on chromosome 4 of *Arabidopsis* and pericentromeres on various chromosomes in addition to that on chromosome 4. This observation suggests that epigenetic mechanisms such as histone modifications and cytosine methylation may also modulate large-scale interactions in the genome. Since plant species are known to adopt different interphase chromosome configurations that may be correlated with genome sizes and/or ploidy levels (Dong and Jiang, 1998; Cowan et al., 2001; Santos and Shaw, 2004), whether the organization pattern observed by Grob et al. (2013) can be generalized to other plant genomes remains to be determined.

Using time-lapse fluorescent imaging of four chromatin beacon lines, each containing a single copy of an identical transgene that has been mapped to a 100 kb region adjacent to the nucleolus organizing region (NOR) on *Arabidopsis* chromosome 2, we obtained evidence consistent with a topological domain (Rosin et al., 2008). This proposed domain shares several characteristics with topological domains described in insects and mammals: (1) The size of the domain we observed is similar to the average chromatin domain found in *Drosophila*, which is consistent with the comparable genome size of *Arabidopsis* and *Drosophila*. (2) The chromatin beacon line residing at the domain boundary (CCT432) appeared much closer to the NOR in 3-D compared to the other three beacons in the proposed domain. The result suggests that association with the nucleolus helps to create and/or maintain the topological domain. This model is akin to observed cases in which topological domain boundaries overlap with lamina-associated domains. (3) The reporter gene in line CCT432, as well as the flanking genes surrounding the transgene in this line, is actively expressed. This is consistent with the observation that housekeeping genes are enriched in topological domain boundaries.

## THE REGULATORY FUNCTION OF CHROMATIN ORGANIZATION

In animal systems, enhancers are distal regulatory elements that can locate from several kb to over one Mb away from the promoter that is being modulated. Enhancers are typically marked by H3K4me1 and H3K27Ac and are depleted of H3K4me3 (Creyghton et al., 2010). Subsets of enhancers are also bound by the co-activator CBP/P300 and/or mediator components (Visel et al., 2009). Enhancers can regulate distant promoters through creating spatial proximity by looping, with involvement of the Cohesin machinery (Kagey et al., 2010). Studies with RNA polymerase II centered ChIA-PET and Hi-C experiments have revealed that distinct cell types can display different subsets of enhancer–promoter interactions (Zhang et al., 2012; Kieffer-Kwon et al., 2013). Mirroring the finding from Hi-C assays that short-range looping is not affected by cellular stimulations (Jin et al., 2013), activating the TNF-α pathway in human fetal lung fibroblast cells (IMR90) does not cause significant remodeling of enhancer-promoter contacts. Therefore, cell-type-specific signaling programs may be established by chromatin architectural features found in particular cell types.

In contrast to the case in mammalian systems, evidence supporting gene regulation by distal elements as a prominent mechanism in plants is scarce. Enhancer traps have been successfully applied in plant systems for the establishment of cell-type-specific marker lines (Haseloff, 1999). However, the responsible regulatory sequences are rarely cloned and analyzed with respect to distant regulatory element – promoter pairs.

A number of recent studies in plants using both proximity ligation and chromatin beacon strategies have suggested chromatin organizations may regulate gene functions for specific pathways. The characterization of regulatory elements at the maize *booster* (b1) locus provided the most convincing example of a plant distal regulatory element to date. Among many alleles of the b1 locus, B–I and B′ were found to contain identical DNA sequences but are associated with distinct chromatin and expression characteristics and were therefore considered epi-alleles (Louwers et al., 2009). The repressed B′ allele was further found to be paramutagenic, which is capable of converting the actively expressed B–I to an inactive B′ allele when combined genetically. A hepta-repeat locating ∼100 kb upstream of the b1 transcription start site (TSS) fits the classical definitions of a distal enhancer element: (1) The presence of multiple 853 bp repeats was required for activated b1 transcription. (2) Using the 3C assay, the hepta-repeats were found to physically interact with the b1 TSS specifically in tissues where b1 is actively expressed. In addition, the authors also found two more restriction fragments each located ∼47 kb and 107 kb upstream that can physically interact with the b1 TSS (Louwers et al., 2009). These two regions interact with the b1 TSS in a manner that correlates with b1 expression levels, suggesting that they may function as novel distal regulatory elements.

Two recent studies analyzed the chromatin conformation of the *Arabidopsis FLC* locus using the complementary 3C and chromatin-beacon approaches. A physical gene loop was discovered between the 5′- end of *FLC* and a region several 100 base pairs downstream of the *FLC* polyadenylation site (Crevillén et al., 2013). Intriguingly, the gene loop is disrupted at an early stage of vernalization (2 weeks in the cold) and remains disrupted after returning to warm temperatures for 7 days. This “cold memory” behavior of the observed loop suggests it can either be a carrier of epigenetic information or it is responsive to other epigenetic inputs. Using a chromatin beacon strategy, Rosa et al. (2013) investigated the physical interactions of multiple *FLC* alleles in endoreduplicated *Arabidopsis* root cells. Physical clustering of *FLC* alleles was observed in response to vernalization. Similarly to disruption of the *FLC* gene loop, clustering of *FLC* alleles also remains stable at least 7 days after plants were returned to warmer conditions.

## 3-D CHROMATIN ARCHITECTURE AND STEM CELL PLURIPOTENCY

Stem cells are uniquely endowed with the capability of infinite self-renewal and a wide differentiation potential to generate multiple cell types. Plants and animals both rely on stem cell populations for embryogenesis and post-embryonic development. In *Arabidopsis*, the major stem cell populations are maintained in the shoot and root apical meristems (SAM and RAM), respectively. Extrinsic cues (phytohormones, wounding, etc.) and signals from stem cell niches have been shown to modulate stem cell activities and to shape plant development (Sablowski, 2004; Singh and Bhalla, 2006). In terms of intrinsic pluripotency factors in plants, the homeodomain transcription factors WUSCHEL (WUS) and SHOOT MERISTEMLESS (STM), when ectopically expressed, induce cellular reprogramming and ectopic shoot stem cell proliferation (Gallois et al., 2002). In RAM, stem cell population is defined by the longitudinal gradient of PLETHORA (PLT) 1 and 2, as well as the APETALA 2 (AP2) transcription factors, and the radial gradient of the SHORTROOT (SHR) and SCARECROW (SCR) transcription factors (Sabatini et al., 2003; Aida et al., 2004; Galinha et al., 2007). Expression of both PLT and SCR can induce ectopic formation of root stem cells (Aida et al., 2004; Galinha et al., 2007).

Extensive studies have shown how pluripotency transcription factors function together and are regulated by chromatin regulators to control cell identity (Papp and Plath, 2013). It has been hypothesized that transcription regulatory circuits and epigenetic modification patterns are involved in pluripotency induction and stem cell maintenance (Lessard and Crabtree, 2010; Mattout and Meshorer, 2010; Young, 2011). Stem cells can also display distinct features in DNA methylation (Meissner, 2010), histone modification, chromatin remodeling (Chen and Dent, 2014; Thiagarajan et al., 2014), and transcription regulation via non-coding RNA-based mechanisms (Wright and Ciosk, 2013; Bergmann and Spector, 2014). Compared to genomes of differentiated cells, those of stem cells in general have more open chromatin configuration and dynamic association with chromatin proteins, perhaps reflecting their plasticity in self-renewal and pluripotency (Meshorer et al., 2006; Mattout and Meshorer, 2010). The stem cell genomes of mammals are also featured with bivalent chromatin configuration, the co-enrichment of functionally opposite chromatin marks (repressive H3K27me3 and activating H3K4me3) at the TSS of many transcription factors and signaling components (Vastenhouw et al., 2010; Voigt et al., 2013). This promoter “poising” may facilitate more rapid cell fate commitment upon reception of developmental cues by the stem cell populations.

Several recent studies suggested that “form-preceding-function,” where 3-D reorganization of chromatin may occur before detectable transcriptional and phenotypic changes during pluripotency induction. The formation of 3-D chromatin loops by long-range promoter-enhancer associations may coordinate cell type-specific gene expression (Deng et al., 2012; Li et al., 2012; Zhang et al., 2013). In addition, pluripotency factors (Oct4, Nanog, and Sox2) may help to reorganize higher-order chromatin connectivity (Apostolou et al., 2013; de Wit et al., 2013; Ginno and Schubeler, 2013; Krijger and de Laat, 2013) through co-localization and creating long-range contacts in pluripotent stem cells (**Figure 1**). Chromatin architectural proteins (CTCF, Mediator, and Cohesin), in a combinatorial manner, may facilitate the pluripotency factors to bridge cell type-specific chromatin looping (Phillips-Cremins et al., 2013). Indeed, while the overall topologically associating domains (TADs) largely remain unchanged between stem cells and differentiated cells (Dixon et al., 2012; Nora et al., 2012), the genome of pluripotent stem cells displays unique 3-D contacts that are dependent on the expression of pluripotency factors and the chromatin insulators (Apostolou et al., 2013; de Wit et al., 2013; Wei et al., 2013). In addition, recent studies showed that another layer of regulation provided by the PcG (polycomb group) proteins is working to regulate chromatin 3-D organization in mouse stem cells during reprogramming (Denholtz et al., 2013).

## CELL FATE RESETTING IN PLANTS

In *Arabidopsis*, stem cell transcription factors, like pluripotency factors in mammals, are sufficient to induce shoot or root stem cells ectopically. It is tantalizing to speculate that they may also be involved in architectural reorganization of chromatin in the process of cell fate determination in plants. As the topological domains in *Arabidopsis* await to be further established with more 3C and Hi-C studies, here we will discuss recent evidence for linking plant pluripotency factors to epigenetic regulation of stem cell maintenance. Several review articles summarized a wide spectrum of chromatin modifiers and remodelers and their roles in the maintenance of stem cell fate in plants (Kornet and Scheres, 2008; Sang et al., 2009; Shen and Xu, 2009). For example, a histone acetyltransferase AtGCN5 has been shown to regulate both shoot and root stem cells *via* controlling the expression of *WUS* and *PLT*, respectively. Another conserved chromatin remodeler, the SWI/SNF-type ATPase factor, SPLAYED (SYD), was shown to directly bind to the WUS promoter for activation of WUS expression in the SAM (Kwon et al., 2005). The shoot pluripotency factor STM is suppressed by CURLY LEAF (CLF), a PcG protein, and deposition of H3K27me3 marks at the promoter region (Katz et al., 2004; Schubert et al., 2006). A recent study demonstrated that *CLF* also suppresses stem cell resetting in stomatal development (Lee et al., 2014). Mature guard cells (GCs), a terminal cell type in the epidermis, are once thought to be irreversibly differentiated. A recent study showed that cell fate of mature GCs can be reset by expression of two transcription factors, FAMA and FOUR LIPS (FLP), resulting in a stoma-in-stoma phenotype. Constitutive expression of *CLF* abrogated this cell fate resetting, apparently by silencing the stomatal stem cell genes SPEECHLESS (SPCH) and MUTE, which encode bHLH transcription factors (Lee et al., 2014). These genetic studies in plant development indicate the potential involvement of chromatin modifiers/regulators in early steps of cell fate specification. Whether genome architecture *per se* is a driver for this developmental commitment is a key question that needs to be resolved.

## FUTURE DIRECTIONS

**FIGURE 2:**
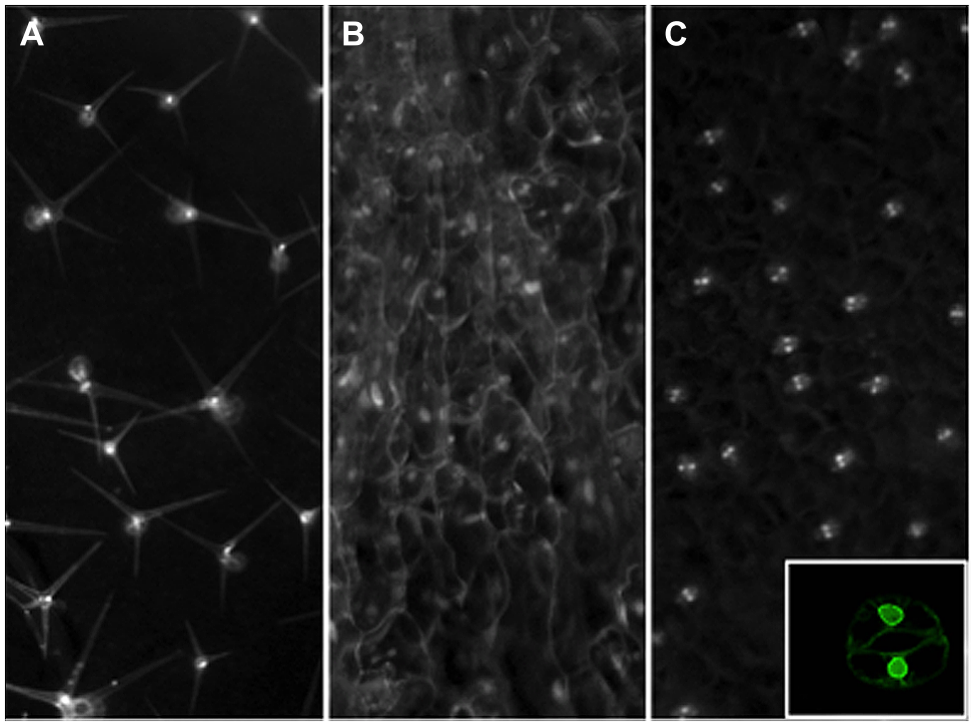
**Nuclear envelope tagging of specific leaf cell types in *Arabidopsis***. Transgenic plants were created with Inserts that express an NTF marker gene driven by the GL2 **(A)**, CaMV35S **(B)**, or AtMYB60 **(C)** promoters for trichome, constitutive, or guard cell-specific expression. The NTF marker is a nuclear envelope-anchored GFP fused to a biotinylation epitope from bacteria. For the GL2 promoter driven construct, a separate vector is used to produce the bacterial *BirA* gene in* trans* to catalyze the biotinylation of the NTF in order to facilitate rapid nuclei purification (Deal and Henikoff, 2010). To create a more facile labeling system, we have generated a new INTACT vector that contains both the NTF expression cassette as well as the *BirA* gene for plant expression. The CaMV 35S promoter and a guard cell-specific promoter from the AtMYB60 gene are used to create the constructs shown in panels **(B)** and **(C)**, respectively. Mature rosette leaves from stable transgenic lines are examined by a stereo epifluorescence microscope fitted with a GFP filter set for images shown. Inset on panel **(C)** shows a confocal image of a tagged guard cell from a single focal plane to Illustrate the nuclear envelope localization of the expressed NTF protein from the new vector driven by the AtMYB60 promoter.

While the 3C-derived chromatin proximity assay and chromatin beacon platform in *Arabidopsis* open up opportunities to close the physical scale gap for studies on chromatin architecture and function, the coexistence of multiple cell types is a confounding issue that often limits the interpretations that can be derived. A facile method for rapid purification of intact nuclei from specific cell types at different stages will greatly augment these techniques by providing additional specificities. The INTACT (isolation of nuclei tagged in specific cell types) method was demonstrated to enable production of cell-type-specific ChIP-seq in *Arabidopsis* root cells (Deal and Henikoff, 2010), as well as nucleosome occupancy maps for muscle cells of mature *Caenorhabditis elegans* and mesoderm cells of *Drosophila* embryos (Steiner et al., 2012). As shown in **Figure 2**, this method can be readily adapted to specifically tag distinct populations of epidermis-derived leaf cell types such as trichomes and GCs in *Arabidopsis*. Using this approach, we can now deploy development time- and cell type-specific promoters to enable the rapid isolation of nuclei from distinct cell types at defined steps of development. The integration of this method with other genome-wide, sequence-based techniques as well as the chromatin beacon resource should narrow the temporal gap in our methods to study the architecture of chromatin at a defined developmental time and space. We are hopeful that this fusion of new methodologies should finally bring us closer to mapping the early changes in chromatin organization that may drive predictable genome outputs and cell fates.

## Conflict of Interest Statement

The authors declare that the research was conducted in the absence of any commercial or financial relationships that could be construed as a potential conflict of interest.
